# The Pretesting Effect: Exploring the Impact of Feedback and Final Test Timing

**DOI:** 10.5334/joc.455

**Published:** 2025-07-22

**Authors:** Yeray Mera, Nataliya Dianova, Eugenia Marin-Garcia

**Affiliations:** 1 University of the Basque Country UPV/EHU, Spain

**Keywords:** Pretesting effect, Feedback timing, Testing, Error learning, Retrieval

## Abstract

The pretesting effect suggests that attempting and failing to guess unknown information can improve memory compared to errorless study. A relevant question concerns the optimal timing for providing corrective feedback and administering the final test. This study explored two variables: (1) the timing of feedback after unsuccessful pretest attempts, either immediately or following a delay of 24 hours (Experiment 1) or 48 hours (Experiment 2); and (2) the timing of the final test after feedback, either immediately or after a 24-hour delay (Experiment 1). Recall accuracy was evaluated across these conditions and compared to an errorless (read-only) learning condition. The results showed that pretesting consistently yielded higher recall accuracy than the read-only condition. Immediate feedback was more effective than delayed feedback, and performance on the immediate test was superior to that of the delayed test. More importantly, the pretesting effect persisted even with delays in feedback and final testing. This flexibility in timing suggests practical applications, particularly in educational settings where immediate feedback or testing may not always be feasible.

Learning is a fundamental cognitive process that shapes our understanding of the world and our ability to navigate it effectively. For decades, educators and cognitive scientists have debated the most effective strategies for acquiring and retaining knowledge, with a long-lasting focus on the outcomes of errorful versus errorless learning. Proponents of errorless learning have argued for protecting learners from encountering errors during the learning process, aiming to prevent the reinforcement of incorrect information in memory and to avoid repeating those errors in the future (e.g., Ausubel et al., 1969; Bandura, 1986; Skinner, 1953, 1958). However, this approach also deprives learners of valuable learning opportunities that arise from receiving corrective feedback after making errors. Contrary to this traditional view, one of the most intriguing and counterintuitive findings in recent years is that making errors during the learning process can enhance memory. A growing body of evidence suggests that errorful learning, far from being detrimental, can be beneficial and even foster deeper learning (for a review, see Mera et al., 2022; Metcalfe, 2017). This counterintuitive finding challenges traditional views of errorless learning and opens up new possibilities for educational practices and memory improvement strategies.

This shift in thinking closely aligns with long-standing debates in educational psychology regarding the role of errors in the learning process. The consequences of errors for learning depend on how they are addressed. Rather than being inherently detrimental, errors can create valuable learning opportunities. In discovery learning, for instance, learners generate solutions before receiving formal instruction (Giebl et al., 2021; Kuhn et al., 2000; Zhang & Fiorella, 2023). However, corrective feedback is critical for the effectiveness of these approaches (Butler et al., 2008). This concept is central to productive failure, wherein initial errors lead to a deeper understanding when followed by structured guidance (Kapur, 2008; Loibl et al., 2017; Loibl & Leuders, 2019).

Within this broader framework, one of the most compelling examples of the benefits of errorful learning is the *pretesting effect*. This well-established phenomenon shows that strategically testing individuals on unknown information before they are exposed to it can significantly enhance long-term memory retention (Bartl et al., 2024; DiMarco, et al., 2024; Hartley, 1973; Hollins et al., 2023; Mera, Migueles et al., 2025; Overoye et al., 2021; Potts & Shanks, 2014; Richland et al., 2009; Seabrooke et al., 2019, 2021; Tanaka et al., 2019; Zawadzka & Hanczakowski, 2019). This finding suggests that even when retrieval attempts during a pretest are unsuccessful, including corrective feedback can still lead to improved memory recall compared to traditional errorless study methods, such as simply reading the material for an equivalent amount of time.

A compelling example of the pretesting effect is demonstrated in the study by Kornell et al. (2009). Their research employed a pretesting procedure where participants were unlikely to answer correctly, using weakly related word pairs that participants had not previously encountered. In this paradigm, participants were first presented with a cue word (e.g., pond –?) and asked to guess the target word, after which they received corrective feedback (e.g., pond–frog). To ensure the results reflected the effect of unsuccessful retrieval attempts, any correctly guessed items were excluded from the analysis. Remarkably, the results consistently revealed a clear advantage of pretesting over a read-only condition. This benefit was observed even with the use of trivia questions and when the final test was delayed. These findings are particularly striking given that the pretest trials generated many errors yet still led to improved recall performance.

Furthermore, other related phenomena support the idea that active engagement during learning enhances long-term retention. A well-established example is the Testing Effect, also known as Retrieval Practice (see Roediger & Karpicke, 2006a, for a review; and Rowland, 2014, for a meta-analysis). This effect refers to the robust memory benefit of actively retrieving previously studied information, which not only strengthens memory for the retrieved content (backward effect; Roediger & Butler, 2011) but can also enhance the encoding of subsequently studied material (forward effect; Pastötter & Bäuml, 2014). Retrieval practice engages a distinct neural network (Marin-Garcia et al., 2021; Van den Broek et al., 2016) and is widely considered as one of the most effective learning strategies (Dunlosky et al., 2013). Although similar in spirit, pretesting and retrieval practice differ in their implementation: pretesting involves attempting to answer questions before studying the material, whereas retrieval practice requires recalling information after initial learning. Both approaches illustrate how retrieval—even if initially unsuccessful—can serve as a powerful mechanism for promoting durable learning.

Although both pretesting and retrieval practice employ testing as a learning tool, they differ theoretically in terms of timing and their main cognitive influence. Pretesting prepares the learner for encoding by activating prior knowledge. It also promotes error generation, which can enhance attention and the processing of subsequent corrective feedback. This preparatory activation and error correction process optimizes how information is initially learned. In contrast, retrieval practice primarily consolidates existing learned information and improves the subsequent retrieval. Thus, the distinct advantage of pretesting lies in its emphasis on optimizing the encoding of new information.

Another key difference lies in the nature of the errors. In pretesting, errors may reflect mere guesses, rather than genuine errors, as there is no prior exposure to the learning materials (Potts & Shanks, 2014). Thus, most responses tend to be incorrect, which may affect learning differently compared to scenarios with a more balanced proportion of correct and incorrect answers. Retrieval practice, in contrast, follows a study phase, allowing for more meaningful retrieval attempts and clearer comparisons between correct and incorrect responses (e.g., Kang et al., 2011). However, retrieval practice may be influenced by item-selection effects. Pretesting helps address this issue by enabling a controlled examination of how errors affect later memory (Kornell et al., 2009).

While the pretesting effect has been consistently demonstrated across various conditions, a key question has emerged regarding the optimal timing for providing corrective feedback and administering the final test. The effectiveness of the pretesting approach may depend on when corrective feedback is given after an error and when the final test is conducted. However, the literature presents mixed findings on the ideal timing for both. For instance, studies report varied results regarding the timing of feedback following an error (e.g., Kornell, 2014; Metcalfe et al., 2009) and the timing of the final test (e.g., Kliegl et al., 2024; Zawadzka et al., 2023). This variability highlights the complexity of the pretesting effect and its sensitivity to temporal factors. Given these inconsistencies, it is essential to determine whether a critical period exists for delivering feedback and conducting the final test to maximize the benefits of the pretesting effect.

## Studies with Delayed Corrective Feedback

The effectiveness of pretesting relies on learners receiving corrective feedback after committing errors, as this feedback facilitates the encoding of correct information (Huelser & Metcalfe, 2012; Metcalfe et al., 2012; Metcalfe & Kornell, 2007). Without feedback, learners are unlikely to spontaneously correct their errors (Butler et al., 2008). Feedback thus serves as a critical learning aid, offering information on response accuracy and guiding learners toward correct answers (Pashler et al., 2005). However, in many educational settings, feedback is often delayed (Knight et al., 2012), raising concerns about how such delays might affect learners’ ability to effectively correct their errors.

The pretesting effect can be interpreted through the lens of prediction error learning. In general, traditional learning theories propose that learning is driven by the discrepancy between an event and prior expectations, with larger discrepancies capturing more attention and leading to enhanced learning (e.g., Rescorla & Wagner, 1972). This concept has been applied to memory research, suggesting that incorrect guesses may enhance the processing of immediate corrective feedback (Grimaldi & Karpicke, 2012). Research further indicates that feedback contradicting expectations captures more attention (e.g., Butterfield & Metcalfe, 2001; Fazio & Marsh, 2009), which aligns with evidence suggesting that errors violating prior expectations can facilitate subsequent correct recall (e.g., Brod, 2021; Brod et al., 2018). Thus, in the pretesting context, the discrepancy between a generated error and the subsequent corrective feedback may act as a learning signal, capturing attention and enhancing the encoding of the correct answer (Metcalfe, 2017). However, delaying corrective feedback may reduce the salience of the prediction error, potentially impairing the learner’s ability to effectively associate the cue with the correct response. Furthermore, it remains unclear whether a critical time window exists for providing feedback, beyond which its effectiveness significantly declines.

The prevailing view suggests that pretesting is most effective when corrective feedback is provided immediately after an error is committed (e.g., Grimaldi & Karpicke, 2012; Hays et al., 2013; Vaughn & Rawson, 2012). However, this remains a topic of debate, with some studies reporting mixed results (e.g., Kornell, 2014; Metcalfe et al., 2009; Richland et al., 2009; Zawadzka et al., 2023). For instance, Kornell (2014) conducted a series of pretesting experiments and found that generating errors with more complex and meaningful material (e.g., trivia questions) improved recall accuracy even when corrective feedback was delayed. In contrast, less meaningful material (e.g., word pairs) did not yield the same benefit. The author attributed this difference to the activation of richer semantic networks, which fosters more elaborative retrieval processes (Collins & Loftus, 1975). This explanation aligns closely with Carpenter’s (2009, 2011) elaborative retrieval hypothesis, which proposes that retrieval attempts activate semantically related concepts. This activation reinforces the correct response upon receiving feedback and strengthens the associative link between the cue and the target (see also Mera et al., 2022; Grimaldi & Karpicke, 2012).

Similarly, the impact of feedback timing on the effectiveness of retrieval practice or testing effect has been a subject of debate. A meta-analysis by Rowland (2014) found that both immediate and delayed feedback can enhance the testing effect, with delayed feedback yielding a larger effect. In certain cases, delayed feedback may prove even more effective than immediate feedback, as it allows for additional retrieval attempts and introduces spacing, which enhances learning (Butler et al., 2007). However, other studies suggest that immediate feedback can increase the benefits of retrieval practice by enabling learners to quickly correct errors and reinforce correct responses (e.g., Aljabri, 2024; Li, 2020; Lu et al., 2021), while some research finds no significant difference between delayed and immediate feedback (e.g., Iwaki et al., 2017). Additionally, Kulhavy (1977) noted that delayed feedback might be less effective when participants are unable to provide any response, particularly in difficult tasks or those involving unfamiliar information, as often occurs in pretesting.

One of the few studies that specifically examined delayed feedback in pretesting is by Zawadzka et al. (2023). They conducted two experiments to explore how pretesting benefits emerge when using semantically related word pairs as study material and providing delayed feedback. To increase the richness of the study materials, they employed *context reinstatement*, presenting the word pairs with unrelated context images (e.g., Godden & Baddeley, 1975). In Experiment 1, participants studied semantically related word pairs in Polish, displayed over unrelated photographs. The study phase included three conditions: read-only, immediate feedback, and delayed feedback (with an average delay of 50 trials between guessing and feedback in the delayed condition). During the final test, some items were presented with original study-phase contexts (reinstated-context condition), while others were shown with novel contexts. The results revealed that pretesting significantly improved memory compared to the read-only condition, even with delayed feedback. Notably, the pretesting benefit persisted regardless of whether contexts were reinstated or novel.

Experiment 2 built on Experiment 1 by removing the context from the final test and introducing a delayed-feedback novel-context condition. Participants were tested under four conditions: read-only, immediate feedback, delayed feedback with reinstated context, and delayed feedback with novel context. The results were consistent with those of Experiment 1, indicating that pretesting with delayed feedback improved memory for related word pairs. However, this effect was observed only when feedback was provided in the same context as during the study phase. This finding challenges the notion that immediate feedback is essential for observing the pretesting effect.

## Studies with a Delayed Final Test

The timing of the final test may also play a crucial role in the effectiveness of the pretesting effect. However, it remains unclear whether the interval between the learning phase—during which errors occur and feedback is provided—and the final test impacts subsequent retrieval. Classic studies (e.g., Ebbinghaus, 1964) established that the rate at which information is forgotten is influenced by various factors, including the time since initial learning, and follows a logarithmic pattern (a finding later replicated by Murre & Dros, 2015). However, it remains uncertain whether the length of the retention interval affects the process of error correction and its impact on memory retrieval.

Several studies have shown that the benefits of pretesting persist even after a retention interval of at least 24 hours, using materials such as word pairs (e.g., Kornell et al., 2009; Richland et al., 2009; Vaughn et al., 2017) and trivia questions (Kornell, 2014). However, the influence of retention interval length on the *magnitude* of the pretesting effect remains unclear. On one hand, immediate testing may lead to insufficient time for consolidation, potentially weakening the effect. On the other hand, when the final test is delayed, learners may have greater difficulty distinguishing their initial errors from the correct answers provided during feedback, increasing the risk of interference or error repetition. Thus, while both very short and extended intervals present distinct challenges, their specific impact on the durability of the pretesting effect is still a matter of debate.

Research on a related phenomenon, the retrieval practice or testing effect (e.g., Roediger & Karpicke, 2006a), offers a different pattern. Studies have consistently shown that the effect becomes more pronounced as the delay between practice and final testing increases. Consequently, the advantage of retrieval practice over re-reading is typically greater on delayed tests (e.g., Bahrick & Hall, 2005; McDaniel et al., 2011; Roediger & Karpicke, 2006b; Toppino & Cohen, 2009). In this regard, Latimier et al. (2019) directly compared pretesting to retrieval practice and found that while both methods improved performance, the retrieval practice effect was significantly larger than the pretesting effect after a one-week retention interval. However, no significant differences were observed when the final test was administered on the same day, whether using text passages (Pan & Sana, 2021) or weakly related word pairs (Mera et al., in preparation).

Given these observations, one might expect that the magnitude of the pretesting effect, similar to the retrieval practice effect, also increases with longer retention intervals before the final test. This phenomenon was explored in depth by Kliegl et al. (2024) in two experiments. In the first experiment, participants studied semantically related word pairs. Half of the cue words were presented with their target words for 10 seconds (e.g., plate – fork; study condition), while the other half were presented alone for 5 seconds, during which participants had to guess the target word. Afterward, the cue and the correct target word were presented for 5 seconds (pretest condition). Participants then completed the final retention test after delays of 1 minute, 10 minutes, or 30 minutes. The results showed that the pretest condition consistently outperformed the study-only condition, with the pretesting effect becoming more pronounced at 10- and 30-minute retention intervals. Furthermore, when using more educationally relevant study material –prose passage texts– and extending the retention interval to one week, pretesting led to better final recall than the study-only condition, with superior performance observed at both the 30-minute and one-week intervals.

In summary, these findings suggest that the benefits of pretesting become more apparent as retention intervals increase. However, it is important to distinguish between within-day intervals (1 to 30 minutes) and longer, multiple-day intervals (e.g., one week). The results for retention intervals exceeding one day were based exclusively on prose passage materials, which differs from the word-pair paradigm typically employed in pretesting studies, including our own. This distinction highlights the need for further research to determine whether the pretesting effect for longer intervals generalizes across different types of learning materials. Moreover, while both pretesting and retrieval practice involve active engagement and retrieval-related processes, retrieval practice tends to show more consistent benefits over longer retention intervals. This may be partly due to traditional retrieval practice paradigms including multiple study and test repetitions (e.g., Roediger & Karpicke, 2006a), which may enhance consolidation processes. In contrast, pretesting typically involves a single test prior to study. In the present study, we equated the number of study and test phases across conditions to better isolate the effects of each learning strategy.

## The Present Study

This experiment aims to address critical gaps in our understanding of the pretesting effect by investigating the role of two key temporal factors: (1) The timing of providing corrective feedback after an unsuccessful initial pretest phase, provided immediately or after a delay of 24 hours (Experiment 1) and 48 hours (Experiment 2), and (2) The timing of the final test after the provision of corrective feedback, either immediately or after 24 hours (Experiment 1). Our primary objective is to determine how these temporal variables and their interaction influence recall accuracy compared to an errorless learning (read-only) condition.

By manipulating these timing factors, we seek to extend the existing literature on pretesting in several important ways: (a) Feedback Timing: While some studies suggest immediate feedback is crucial for the pretesting effect (e.g., Grimaldi & Karpicke, 2012), others have found benefits with delayed feedback, particularly for more complex materials (Kornell, 2014; Zawadzka et al., 2023). Our study aims to shed light on these mixed findings and determine if there is indeed a critical time window for effective error correction, using controlled learning materials such as weak-semantically related word pairs. (b) Final Test Timing: Building on the work of Kliegl et al. (2024), who found that the pretesting effect tends to intensify with longer retention intervals using prose passage texts, our study will explore whether this effect is also observed with simpler and more controlled learning materials, such as word pairs. While Kliegl et al.’s findings for retention intervals exceeding one day were based exclusively on prose passage texts, we will examine whether the benefits of delaying the final test beyond one day also apply to simpler materials. This analysis will help determine whether the pretesting effect, similar to the retrieval practice effect, becomes more pronounced over time for different types of learning materials. (c) Interaction Effects: By systematically varying both feedback and final test timing, we can examine potential interaction effects between these factors. Understanding how these variables interact may reveal the most effective combinations of feedback and test timing for enhancing learning outcomes and maximizing the benefits of pretesting. We also measured other important aspects, such as error types, reaction times and metacognitive judgements, across both experiments. Through this comprehensive approach, we aim to provide a deeper understanding of the temporal dynamics underlying the pretesting effect, going beyond examining these factors in isolation.

## Experiment 1

### Method

#### Participants

Sixty-four students from the University of the Basque Country UPV/EHU were randomly allocated to two groups: The pretest group (n = 32, 31 females, one male, *M*_age_ = 18.31, *SD*_age_ = 0.54) and the read group (n = 32, 24 females, six males, *M*_age_ = 19.72, *SD*_age_ = 8.66).

An *a priori* power analysis indicated that a sample size of 30 participants per group would provide 86% statistical power to detect a large effect of f = .40 for a mixed 2 × 2 × 2 ANOVA. This effect size was selected based on previous studies reporting robust pretesting effects with similar materials and designs (e.g., Kornell et al., 2009; Huelser & Metcalfe, 2012). This power analysis was calculated using G* Power (Faul et al., 2007). To ensure a full counterbalanced dataset, an additional four participants were recruited, resulting in a total of 64 participants for the experiment.

The sample primarily consisted of Psychology students. All participants confirmed being native Spanish language users and reported no diagnosed developmental disorders (e.g., ADHD, Dyslexia), psychological or psychiatric disorders, or use of medication that could affect task performance. Participants provided informed consent to participate and received course credit as compensation. Ethical approval for this study was granted by the Ethics Committee for Research and Teaching (CEID/IIEB) of the University of the Basque Country UPV/EHU (M10-2019-152).

#### Design

The experiment employed a 2 × 2 × 2 mixed factorial design, which included one between-subjects factor with two levels (learning condition: pretest, read) and two within-subjects factors, each with two levels: feedback (immediate, delayed) and final test (immediate, delayed). The decision to manipulate the learning condition between subjects was made to prevent potential contamination effects, such as carryover effects that might arise if participants experienced both learning methods within the same session. This approach is consistent with prior research on pretesting (e.g., Grimaldi & Karpicke, 2012). Both the assignment of item lists to feedback and test conditions, as well as the order in which participants completed these conditions, were fully counterbalanced across participants (see Table 1).

**Table 1:** Three-Day Timeline of the Design Used in Experiment 1.


GROUP	CONDITION	DAY 1	DAY 2	DAY 3

Pretest	IF-IT	Pretest (8s) + F (5s) – Final test	—	—

	IF-DT	Pretest (8s) + F (5s)	Final test	—

	DF-IT	Pretest (8s)	F (5s) – Final test	—

	DF-DT	Pretest (8s)	F (5s)	Final test

Read	IF-IT	Read (13s) – Final test	—	—

	IF-DT	Read (13s)	Final test	—

	DF-IT	Read (8s)	Read (5s) – Final test	—

	DF-DT	Read (8s)	Read (5s)	Final test


*Note*. “IF-IT” indicates immediate feedback followed by an immediate test, “IF-DT” indicates immediate feedback followed by a delayed test, “DF–IT” indicates delayed feedback followed by an immediate test, and “DF–DT” indicates delayed feedback followed by a delayed test.

#### Materials

The study materials comprised 80 weakly semantically related Spanish word pairs (e.g., mouse–computer), with 20 pairs allocated to each within-subject condition. These pairs were chosen from the NALC database (Fernández et al., 2010). Following Kornell et al.’s selection criteria (2009), pairs were chosen with a forward cue-to-target strength between .05 and .054, meaning that the target word was the first association for 5% of the participants when presented with the cue. All words were nouns with a minimum of four letters and scored higher than 4 on measures of familiarity, imaginability, and concreteness (EsPal repository, Duchon et al., 2013). Each set of 20 word pairs was matched to one of the four learning conditions.

#### Procedure

**Figure 1:**
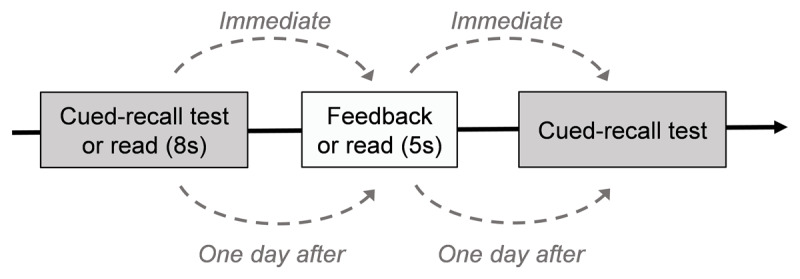
Schematic Representation of the Procedure Used in Experiment 1. *Note*. Participants began with either a cued-recall pretest or a reading session of the word pairs, followed by corrective feedback (or extended exposure to the pairs) delivered either immediately or after a one-day delay. They then completed a final cued-recall test either immediately after receiving feedback (or extended reading), or following a one-day delay.

Over three consecutive days, participants were randomly assigned to one of the two learning groups (pretest or read) and completed three experimental phases: the learning phase, distractor task phase, and test phase (See Figure 1). In addition, three-word pair practice trials preceded each phase, and participants were encouraged to address any questions they might have during that time. There were four experimental conditions: Immediate Feedback – Immediate Test (IF-IT), Immediate Feedback – Delayed Test (IF-DT), Delayed Feedback – Immediate Test (DF-IT), and Delayed Feedback – Delayed Test (DF-DT).

**Day 1**. During the *learning phase*, participants in the pretest group were shown cue words (e.g., mouse–?) and were asked to guess the target word within 8 seconds. Corrective feedback (e.g., mouse–computer) was provided for 5 seconds. For half of the word pairs, feedback was provided immediately after their response (IF-IT and IF-DT conditions). In contrast, participants in the read group read half of the word pairs for a total of 13 seconds and the other half for 8 seconds.

Following the learning phase, participants completed the *distractor task phase*, involving a five-minute Continuous Performance Test. In this task, participants were instructed to press the space bar only when a specific target letter (e.g., “K”) appeared among a rapid succession of letters, refraining from responding to other letters. Finally, participants proceeded to the *final test phase*. In this phase, the pretest group completed a cued recall test for half of the word pairs that had received immediate feedback, while the read group was tested on half of the word pairs they had studied for 13 seconds (IF-IT condition). During this phase, participants were presented with cues (e.g., mouse–?) from the study phase and asked to correctly recall the targets. No corrective feedback was provided during this phase. The test was not time-limited, and participants could leave answers blank (i.e., omission errors).

**Day 2**. This began with delayed corrective feedback. For the pretest group, this involved the presentation of the remaining half of word pairs not revealed after pretesting on Day 1 (DF-IT and DF-DT conditions). For the read group, this phase consisted of presenting the word pairs for an additional 5 seconds, completing the total presentation time for pairs initially read for only 8 seconds on Day 1. Following this, participants completed the five-minute *distractor task*. The day concluded with the *final test phase*, which consisted of a cued recall test for the remaining pairs not tested on Day 1 (IF-DT condition) and for half of the pairs that received corrective feedback (or extended reading time) on Day 2 (DF-IT condition).

**Day 3**. This day began directly with a cued recall test of the remaining pairs that had not yet been tested (DF-DT condition). Upon completion of the experiment, participants were debriefed and thanked for their participation.

The experiment was conducted in a quiet room with no distractions. The material was presented in white text on a black background on a 1680 x 1050-resolution computer. The open-source software “PsychoPy” (v2022.2.5) was used for stimulus presentations and data collection (Peirce et al., 2019), which enables precise recording of reaction times (Bridges et al., 2020). The entire experiment lasted approximately 40 minutes (Day 1: 20 m, Day 2: 10 m, and Day 3: 5 m).

**Metacognitive Judgments**. After each of the four final tests, participants were asked to predict how much of the material they had answered correctly, providing an estimate ranging from 0% to 100%. Following the final test on Day 3, participants also rated the perceived effectiveness of each of the two learning conditions on a scale ranging from 1 (not at all effective) to 7 (extremely effective).

### Results

#### Data Analysis

All analyses were conducted using RStudio (R CoreTeam, 2023). To complement traditional null hypothesis significance testing (NHST), we also computed Bayes Factors (BF) to quantify the strength of evidence for the alternative hypothesis (H_1_) relative to the null hypothesis (H₀). Bayes Factors were calculated using the *BayesFactor* package (version 0.9.12–4.7; Morey & Rouder, 2023). Interpretations of BF values followed the classification scheme originally proposed by Jeffreys (1961) and later elaborated by Lee and Wagenmakers (2013), which provides categorical labels (e.g., anecdotal, moderate, strong) to guide interpretation of the strength of evidence.

#### Scoring

Responses provided by participants during the initial and final cued-recall tests were recorded and evaluated for accuracy. The computer program automatically scored responses as correct if the typed word matched the correct target. Before analyzing the data, typographical errors were reviewed. Minor errors (observed in some cases), such as incorrect accenting (e.g., “raton” vs. “ratón”), were coded as correct. The scoring program also converted all responses to lowercase to ensure consistency. During data processing, responses with accidental spaces (e.g., “ratón_” or “ra_tón”) were identified, the spaces were removed, and accuracy was re-evaluated for these trials.

#### Cued recall performance

Given that our main interest was to study the effect of pretested erroneous responses, any items correctly guessed during the learning phase were removed from subsequent analyses, which accounted for 5.63% of the pretested trials. This exclusion is standard practice in pretest research (Kornell et al., 2009). A 2 (learning group: pretest, read) × 2 (feedback: immediate, delayed) × 2 (test: immediate, delayed) mixed Analysis of Variance (ANOVA) revealed significant main effects of learning group, *F*(1, 62) = 29.05, *p* < .001, \[\eta _g^2\] = .12, BF_10_ > 100, with the pretest group (*M* = 60.86%, *SD* = 21.64%) performing better than the read group (*M* = 48.24%, *SD* = 19%), *F*(1, 62) = 29.05, *p* < .001, \[\eta _g^2\] = .12, BF_10_ > 100. A significant main effect of feedback timing was also observed, *F*(1, 62) = 13.40, *p* = .001, \[\eta _g^2\] = .04, BF_10_ = > 7.47, indicating that immediate error correction (*M* = 58.16%, *SD* = 22.03%) was more beneficial than delayed feedback (*M* = 50.94%, *SD* = 19.94%). The effect of test condition was also significant, *F*(1, 62) = 78.88, *p* < .001, \[\eta _g^2\] = .25, BF_10_ > 100, showing that performance was better when the final test was conducted immediately (*M* = 64.32%, *SD* = 20.08%) rather than after a delay (*M* = 44.78%, *SD* = 17.72%). While there was an interaction between feedback and test timing, *F*(1, 62) = 5.94, *p* = .018, \[\eta _g^2\] = .02, BF_10_ = 3.36, no interaction was observed between learning condition and feedback, *F*(1, 62) = 3.52, *p* = .065, \[\eta _g^2\] = .01, BF_10_ = 0.85, nor between learning condition and test timing, *F*(1, 62) = 3.03, *p* = .087, \[\eta _g^2\] = .01 BF_10_ = 1.92. The three-way interaction was also not significant, *F*(1, 62) = 0.30, *p* = .59, \[\eta _g^2\] < .01, BF_10_ = 0.29.

**Figure 2:**
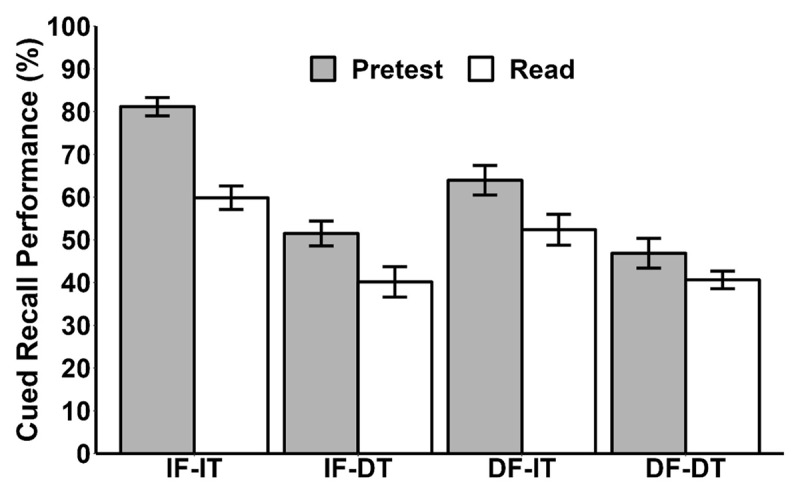
Mean Percentage of Correct Responses on each Cued-Recall Test of Experiment 1. *Note*. “IF-IT” refers to immediate feedback followed by an immediate test. “IF-DT” indicates immediate feedback followed by a delayed test, “DF–IT” refers to delayed feedback followed by an immediate test, and “DF–DT” indicates delayed feedback followed by a delayed test. Error bars represent the standard error of the mean.

Although the three-way interaction was not significant, we conducted exploratory follow-up comparisons to examine performance differences between the pretest and read groups across the four combinations of feedback and test timing. Post hoc comparisons indicated that the pretest group showed significantly higher cued recall accuracy than the read group in the IF-IT, IF-DT, and DF-IT conditions, *t*(62) = 6.12, *p* < .001, *d* = 1.53, BF_10_ > 100, *t*(62) = 2.47, *p* = .016, *d* = 0.62, BF_10_ = 3.19, and *t*(62) = 2.31, *p* = .024, *d* = 0.58, BF_10_ = 2.36, respectively. However, no significant difference was found in the DF-DT condition, *t*(62) = 1.54, *p* = .128, *d* = 0.39, BF_10_ = 0.69 (see Figure 2).

To further examine the magnitude of the pretesting effect across feedback and test timing conditions, we calculated Cohen’s *d* with 95% CI (Confidence Intervals). Interestingly, we observed a decrease in the magnitude of the pretesting effect when delaying the timing between the error and corrective feedback. For immediate feedback, a large effect was found, *d* = 1.24, *p* <.001, 95% CI [0.69, 1.78], with a smaller effect for delayed feedback, *d* = 0.82, *p* = 002, 95% CI [0.30, 1.34]. The improvement decreased from 16.54% (66.54% vs. 50%) to 8.91% (55.39% vs. 46.48%). However, the difference between the confidence intervals (CIs) was not significant. The pretesting effect showed a similar trend according to the timing of the final test. For immediate testing, a large pretesting effect was observed, *d* = 1.29, *p* <.001, 95% CI [0.74, 1.84], and for delayed testing, the effect was smaller, *d* = 0.69, *p* = .008, 95% CI [0.17, 1.20]. The improvement decreased from 16.46% (72.55% vs. 56.09%) to 8.84% (49.23% vs 40.39%), but again, there was no significant difference in the CIs.

#### Error Type Analysis

We analyzed the types of errors made by participants across the different learning groups, feedback, and test conditions. Four participants from the pretest group were excluded from this analysis because they did not make any errors in at least one condition of one of the final tests. The participants’ errors were categorized into four types: (a) *commission errors*—inadequate or incorrect responses that were different from participants’ errors in the learning phase; (b) *omission errors*—no response given on the final test; (c) *confusions*—responses where participants provided the target of a different cue; and (d) *intrusions*—responses that repeated the same errors that participants made during the learning phase (this error type only applies to the pretest group). Table 2 shows the mean proportions and standard deviations for each error type.

**Table 2:** Proportions of Error Types (Commission, Omission, Confusion, and Intrusion) Across Test Conditions in Experiment 1.


ERROR CATEGORY	IMMEDIATE FEEDBACK				DELAYED FEEDBACK			
	
	IMMEDIATE TEST		DELAYED TEST		IMMEDIATE TEST		DELAYED TEST	
			
	PRETEST	READ	PRETEST	READ	PRETEST	READ	PRETEST	READ

Commission	.63 (.32)	.62 (.26)	.53 (.21)	.70 (.21)	.56 (.30)	.70 (.27)	.53 (.18)	.78 (.18)

Omission	.00 (.02)	.19 (.25)	.02 (.06)	.14 (.21)	.01 (.04)	.16 (.26)	.03 (.10)	.12 (.19)

Confusion	.12 (.17)	.19 (.16)	.17 (.14)	.16 (.14)	.20 (.24)	.14 (.13)	.13 (.11)	.10 (.11)

Intrusion	.24 (.25)	—	.28 (.19)	—	.23 (.18)	—	.31 (.18)	—


*Note*. Intrusion errors—incorrect test responses that repeated learners’ errors generated during the initial study phase—were not applicable to the read group. Standard Deviations are in parentheses.

We conducted 2 × 2 × 2 mixed ANOVAs to examine the effects of learning group (pretest or read), feedback (immediate or delayed), and test timing (immediate or delayed) on the proportion of error types (commission, omission, confusion, intrusion). Regarding **commission errors**, a main effect of learning condition was observed, *F*(1, 58) = 13.34, *p* = .001, \[\eta _g^2\] = .08, BF_10_ = 36.18, and an interaction between learning and test conditions, *F*(1, 58) = 5.56, *p* = .022, \[\eta _g^2\] = .02, BF_10_ = 7.37. Post hoc pairwise *t*-test comparisons revealed that the read group made more commission errors than the pretest group when the test was delayed (*p* < .001), but not when the final test was immediate (*p* = .11). Additionally, there were more commission errors on the delayed than the immediate test for the read group (*p* = .049) but not for the pretest group (*p* = .41). Regarding **omission errors**, a significant effect of learning condition was found, *F*(1, 58) = 13.43, *p* = .001, \[\eta _g^2\] = .14, BF_10_ = 54.79, and an interaction between learning condition and test timing, *F*(1, 58) = 6.13, *p* = .02, \[\eta _g^2\] = .01, BF_10_ = 15.48. Post hoc comparisons revealed that the read group made a higher proportion of omission errors than the pretest group on both the immediate and delayed final tests (both *p* < .001). No significant differences were found when comparing the timing of the final test within groups (*p* > .05). Regarding **confusion errors**, a main effect of test timing was found, *F*(1, 58) = 4.78, *p* = .033, \[\eta _g^2\] = .01, with more confusion errors occurring on the immediate test (*M* = .18, *SD* = .18) than the delayed test (*M* = .14, *SD* = .13), however Bayesian analysis was anecdotal, BF_10_ = 0.72. No other significant effects were found (*p* > .05). Finally, **intrusion errors** were analyzed only for the pretest group, as the read group could not commit this type of error. The analysis indicated no differences in the proportion of intrusion errors according to feedback, *F*(1, 59) = 0.07, *p* = .79, \[\eta _g^2\] < .01, BF_10_ = 0.14, test timing, *F*(1, 59) = 2.75, *p* = .10, \[\eta _g^2\] = .01, BF_10_ = 0.57, or an interaction between the two, *F*(1, 59) = 0.19, *p* = .66, \[\eta _g^2\] < .01, BF_10_ = 0.21, suggesting minimal proactive interference.

#### Reaction Times

Reaction times (RT) on the final test were analyzed by calculating the time (in seconds) between the presentation of the cue and the first keypress of the response. RTs that exceeded two standard deviations from the participant’s mean or were faster than 100 ms were corrected for each participant’s mean. Based on these criteria, 5.88% of observations were corrected. A mixed 2 × 2 × 2 ANOVA revealed a significant main effect of learning condition, *F*(1, 62) = 6.36, *p* = .014, \[\eta _g^2\] = .04, BF_10_ = 2.38, test timing, *F*(1, 62) = 23.84, *p* < .001, \[\eta _g^2\] = .05, BF_10_ > 100, and an interaction between learning condition and feedback timing, *F*(1, 62) = 22.16, *p* < .001, \[\eta _g^2\] = .07, BF_10_ > 100. There was no significant main effect of feedback, *F*(1, 62) = 0.01, *p* = .92, \[\eta _g^2\] < .01, BF_10_ = 0.13, or interaction between learning condition and test, *F*(1, 62) = 0.83, *p* = .365, \[\eta _g^2\] < .01, BF_10_ = 0.26, or feedback and test, *F*(1, 62) = 0.01, *p* = .92, \[\eta _g^2\] < .01, BF_10_ = 0.18. The three-way interaction was also not significant, *F*(1, 62) = 0.35, *p* = .56, \[\eta _g^2\] < .01, BF_10_ = 0.29. Post hoc comparisons were used to analyze the source of the interaction between learning condition and feedback timing. Regarding the pretest group, participants were slower to respond when the feedback was delayed (*M* = 4.03, *SD* = 1.75) compared to when it was administered immediately (*M* = 3.36, *SD* = 1.28), *t*(126) = 2.44, *p* = .02, *d* = 0.43, BF_10_ = 2.75. In contrast, in the read group, participants took longer to respond when the feedback was immediate (*M* = 3.35, *SD* = 1.22) compared to when it was delayed (*M* = 2.85, *SD* = 0.68), *t*(126) = 3.95, *p* < .001, *d* = 0.70, BF_10_ > 100.

#### Metacognitive Judgments

Additionally, participants were asked to predict their performance and rate the effectiveness of each learning condition. A two-way mixed ANOVA was conducted to compare predicted performance with real performance across learning conditions. This analysis revealed an interaction between performance type (real, predicted) and learning conditions (pretest, read), *F*(1, 62) = 10.33, *p* = .002, \[\eta _g^2\] = .04, BF_10_ = 5.36. In the pretest group, participants predicted lower performance (*M* = 51.28%, *SD* = 10.72%) compared to their actual performance (*M* = 60.96%, *SD* = 9.00%), *t*(62) = 3.91, *p* < .001, *d* = 0.98, BF_10_ = 41.55. In contrast, in the read group, no significant difference was found between predicted performance (*M* = 48.51%, *SD* = 17.49%) and real performance (*M* = 48.24%, *SD* = 9.61%), *t*(62) = 0.08, *p* = .94, *d* = 0.02, BF_10_ = 0.14. Additionally, participants in the pretest group rated experiencing errors as beneficial for their learning with a mean of 4.60 points on a 1–7 scale (*SD* = 1.20), which did not differ from the read group (*M* = 4.25, *SD* = 1.08), *t*(62) = 1.21, *p* = .23, *d* = 0.30, BF_10_ = 0.47.

## Experiment 2

Building on the results of Experiment 1, Experiment 2 aimed to investigate whether the pretesting effect persists when corrective feedback is delayed by an extended period of 48 hours. This modification was designed to evaluate the robustness of the pretesting effect when feedback is delayed even further. In contrast to Experiment 1, which manipulated both feedback and test timing, Experiment 2 focused exclusively on feedback timing while administering the final test immediately after feedback. This allowed us to isolate the impact of feedback timing alone, without introducing variation in test delay. This experiment used a within-subjects design to directly compare the learning effectiveness of the pretesting and reading conditions. By adopting this approach, the experiment aimed to gain a clearer understanding of how extended feedback intervals affect error correction, a critical issue for optimizing learning in educational settings where delayed feedback is frequently applied.

### Method

#### Participants

Twenty-five students from the University of the Basque Country UPV/EHU (18 females, seven males, *M*_age_ = 18.40, *SD*_age_ = 1.41) participated in Experiment 2. An a priori power analysis using G* Power (Faul et al., 2007) determined that a sample size of 24 participants would achieve 80% statistical power to detect a large effect (*f* = .40) for a within-subjects 2 × 2 ANOVA. The participant selection criteria were the same as in Experiment 1.

#### Design

The experiment employed a 2 × 2 within-subjects factorial design with a learning condition (pretest, read), and a feedback timing condition (immediate, delayed). The pairing of feedback timing (immediate or delayed) and item lists and the order in which participants completed the learning conditions were counterbalanced (see Table 3).

**Table 3:** Three-Day Timeline of the Design Used in Experiment 2


GROUP	CONDITION	DAY 1	DAY 2	DAY3

Pretest	IF	Pretest (8s) + F (5s) – Final test	—	—

	DF	Pretest (8s)	—	F (5s) – Final test

Read	IF	Read (13s) – Final test	—	—

	DF	Read (8s)	—	Read (5s) – Final test


*Note*. “IF” indicates immediate feedback, and “DF” indicates delayed feedback.

#### Procedure

**Figure 3:**
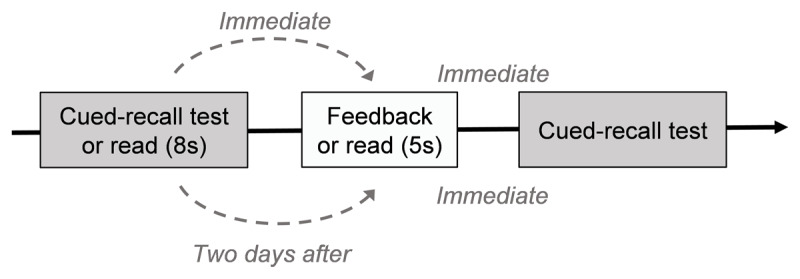
Overview of the Procedure Used in Experiment 2. *Note*. Participants began with either a cued-recall pretest or a reading session of the word pairs, followed by corrective feedback (or extended exposure to the pairs) delivered either immediately or after a two-day delay. They then completed a final cued-recall immediately after receiving feedback (or extended reading).

Experiment 2 differed from Experiment 1 primarily in employing a fully within-subjects design. Participants experienced both learning conditions, with half of the word pairs studied through pretesting and the other half learned by reading. To further examine the effect of feedback timing, half of the pairs received immediate feedback, while for the remaining pairs, this was delayed by two days (compared to the one-day delay in Experiment 1). The timing of the final test was not manipulated and was administered immediately after feedback (see Figure 3).

The experiment was conducted over two days, separated by a one-day break. All participants completed three experimental phases: the learning phase, the distractor task phase, and the test phase. The rest of the procedure was identical to Experiment 1. Participants were debriefed and thanked for their participation upon completion. The entire experiment lasted approximately 40 minutes in total (25 minutes on Day 1, 15 minutes on Day 2). The materials and software used were the same as in Experiment 1.

### Results

#### Cued recall performance

**Figure 4:**
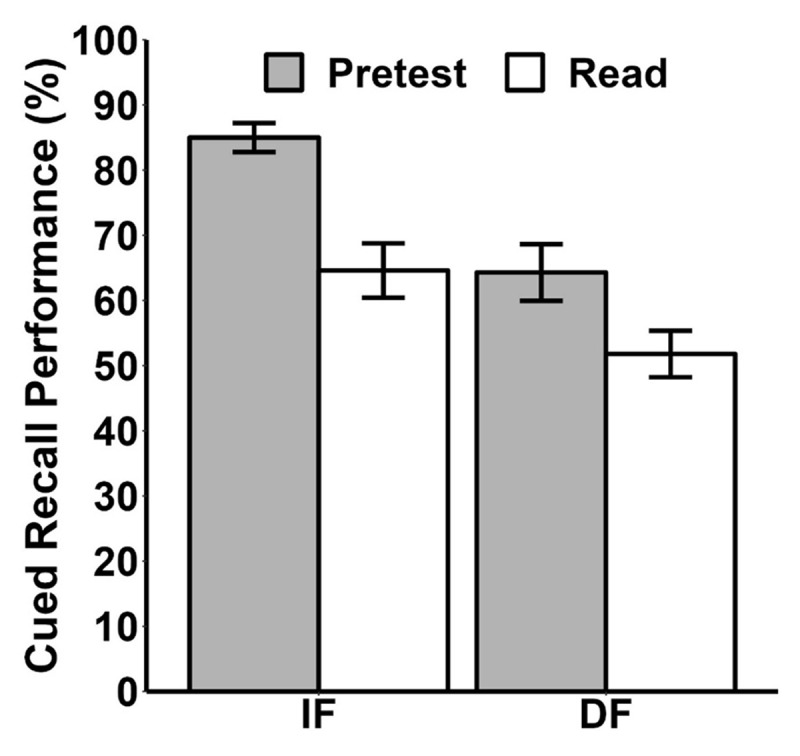
Mean Percentage Correct Responses on each Cued-Recall Test in Experiment 2. *Note*. “IF” refers to immediate feedback, and “DF” refers to delayed feedback. Error bars represent the standard error of the mean.

Participants correctly guessed 6% of the pretested trials, which were excluded from subsequent analysis. A 2 (learning condition: pretest, read) × 2 (feedback: immediate, delayed) within-subjects ANOVA revealed significant main effects of learning condition, with participants performing better in the pretest condition (*M* = 74.64%, *SD* = 20.05%) than the read condition (*M* = 58.20%, *SD* = 20.22%), *F*(1, 24) = 18.81, *p* < .001, \[\eta _g^2\] = .17, BF_10_ > 100, and feedback timing, *F*(1, 24) = 32.31, *p* < .001, \[\eta _g^2\] = .28, BF_10_ > 100, with participants performing better when feedback was delivered immediately (*M* = 74.80%, *SD* = 19.47%) compared to a two-day delay (*M* = 58.04%, *SD* = 20.65%). No significant interaction was found, *F*(1, 24) = 1.22, *p* = .28, \[\eta _g^2\] = .01, BF_10_ = 0.55 (see Figure 4).

Analysis of effect sizes (using Cohen’s *d*) showed a decreasing trend in the magnitude of the pretesting effect with an increase in the interval between the error and corrective feedback. For immediate feedback, the pretesting effect was large, *d* = 1.22, *p* <.001, 95% CI [0.60, 1.84], while for delayed feedback, the effect was smaller, *d* = 0.63, *p* = 03, 95% CI [0.05, 1.21]. The difference in performance (improvement) decreased from 20.39% (84.99% vs. 64.60%) to 12.49% (64.29% vs. 51.80%). However, differences in CIs were not significant.

#### Error Type Analysis

A series of 2 × 2 within-subject ANOVAs were conducted to examine the effects of learning condition (pretest or read), and feedback timing (immediate or delayed) on the proportion of error types (commission, omission, confusion, intrusion). The mean proportions and standard deviations for each error type are presented in Table 4. Three participants were excluded because they made no errors in at least one condition on the final test.

**Table 4:** Proportions of Error Types (Commission, Omission, Confusion, and Intrusion) Across Test Conditions in Experiment 2.


ERROR CATEGORY	IMMEDIATE FEEDBACK		DELAYED FEEDBACK	
	
	PRETEST	READ	PRETEST	READ

Commission	.39 (.36)	.81 (.19)	.47 (.26)	.85 (.14)

Omission	.01 (.04)	.03 (.09)	.04 (.14)	.00 (.00)

Confusion	.27 (.35)	.16 (.18)	.12 (.11)	.15 (.14)

Intrusion	.33 (.36)	*–*	.37 (.30)	*–*


*Note*. Intrusion errors—incorrect test responses that repeated learners’ errors generated during the initial study phase—were not applicable to the read condition. Standard Deviations are in parentheses.

The results revealed a main effect of learning condition on the proportion of **commission errors**, *F*(1, 21) = 53.62, *p* < .001, \[\eta _g^2\] = .40, BF_10_ > 100, with the read condition producing a higher proportion of commission errors (*M* = .83, *SD* = .17) than the pretest condition (*M* = .43, *SD* = .31). However, there was no main effect of feedback timing, *F*(1, 21) = 0.81, *p* = .38, \[\eta _g^2\] = .01, BF_10_ = 0.30, or an interaction between the two variables *F*(1, 21) = 0.25, *p* = .63, \[\eta _g^2\] < .01, BF_10_ = 0.31. Regarding **omission errors**, no main effects of learning condition, *F*(1, 21) = 0.28, *p* = .60, \[\eta _g^2\] < .01, BF_10_ = 0.25, feedback timing, *F*(1, 21) = 0.00, *p* = .97, \[\eta _g^2\] < .01, BF_10_ = 0.22, or an interaction between the two, *F*(1, 21) = 2.29, *p* = .15, \[\eta _g^2\] = .03, BF_10_ = 1.31, were observed. Regarding **confusion errors**, a significant interaction between learning condition and feedback timing was found, *F*(1, 21) = 5.31, *p* = .032, \[\eta _g^2\] = .03. However, the Bayesian analysis indicated this evidence to be anecdotal, BF_10_ = 1.31. Finally, **intrusion errors** were analyzed only for the pretest condition, as the read condition did not allow for this error type. No differences in the proportions of intrusion errors were found between immediate and delayed feedback, *t*(21) = 0.53, *p* = .60, *d* = 0.14, BF_10_ = 0.25, suggesting minimal proactive interference.

#### Reaction Times

RTs exceeding two standard deviations from the participant’s mean or faster than 100 ms were corrected to each participant’s mean. A total of 6.43% of observations were corrected based on these criteria. A within-subjects ANOVA revealed no significant main effect of learning condition, *F*(1, 48) = 0.64, *p* = .43, \[\eta _g^2\] = .01, BF_10_ = 0.44, feedback timing, *F*(1, 48) = 0.81, *p* = .37, \[\eta _g^2\] < .01, BF_10_ = 0.31, or interaction between learning condition and feedback timing, *F*(1, 48) = 2.93, *p* = .093, \[\eta _g^2\] = .02, BF_10_ = 1.01.

#### Metacognitive Judgments

A two-way within-subjects ANOVA revealed an interaction between performance type (real, predicted) and learning condition, *F*(1, 24) = 11.76, *p* = .002, \[\eta _g^2\] = .03, BF_10_ > 100. In the pretest condition, participants predicted lower performance (*M* = 60.48%, *SD* = 18.30%) than their actual performance (*M* = 74.47%, *SD* = 13.01%), *t*(24) = 5.14, *p* < .001, *d* = 0.67, BF_10_ > 100. In contrast, in the read condition, predicted performance (*M* = 56.22%, *SD* = 19.97%) and real performance (*M* = 58.20%, *SD* = 15.32%) were not statistically different, *t*(24) = 0.62, *p* = .54, *d* = 0.09, BF_10_ = 0.19. Additionally, participants in the pretest condition rated that experiencing errors was beneficial for their learning with a mean of 5.00 points on a 1–7 scale (*SD* = 0.87), which did not differ from the read condition (*M* = 4.72, *SD* = 1.28), *t*(24) = 0.91, *p* = .37, *d* = 0.26, BF_10_ = 0.31.

## General Discussion

The present study explored how the timing of corrective feedback and final testing influences the pretesting effect. The findings of this study extend our current understanding of pretesting as a learning strategy, particularly in contexts where feedback and testing may be delayed. While immediate feedback generally results in better recall, our results showed that the pretesting effect persisted even when feedback was delayed by 24 or 48 hours. This challenges the assumption that immediate feedback is essential (e.g., Grimaldi & Karpicke, 2012; Hays et al., 2013; Vaughn & Rawson, 2012) and supports the notion that delayed feedback can still promote learning (e.g., Kornell, 2014; Zawadzka et al., 2023).

The present findings also align with principles from associative learning literature, which emphasizes the importance of temporal contiguity in the formation of associations between stimuli (e.g., Thorndike, 1911; Spencer, 1855; for a review, see Boakes & Costa, 2014). When corrective feedback is provided immediately after an error, the temporal proximity may strengthen the association between the cue and the correct response. In contrast, delayed feedback introduces a temporal gap that can reduce the effectiveness of associative binding, potentially weakening the encoding of the correct information. Thus, this framework provides a useful lens for understanding why immediate feedback led to better performance in our experiments and supports the broader notion that immediate error correction enhances learning.

However, we also observed that delaying feedback slightly reduced the magnitude of the pretesting effect. These findings expand our understanding of the pretesting effect and contribute to the broader debate on the optimal timing for feedback in pretesting. While immediate feedback is beneficial, delayed feedback does not hinder the pretesting benefit. This suggests pretesting as a flexible and practical approach in educational settings where immediate feedback may not be feasible.

The effectiveness of delayed feedback in our study may be explained by the *elaborative retrieval hypothesis* (Carpenter, 2009, 2011), which posits that retrieving information activates semantically related concepts, creating a network of associations that aids later recall. When learners attempt to retrieve information during pretesting, they likely activate semantically related concepts. This activation may persist over time, enabling effective encoding of the correct information even when feedback is delayed. The error detection literature (e.g., Butterfield & Mangels, 2003) also provides valuable insights into how learners identify and process their errors, highlighting the attentional mechanisms that may support error correction during feedback. Furthermore, research on memory updating (Atienza et al., 2011; Kowialiewski & Majerus, 2020; Walker & Hulme, 1999) offers perspectives on how existing memories are modified when new, potentially conflicting information is encountered. This process is particularly relevant to understanding how pretesting facilitates the application of corrective feedback to address errors.

Regarding the timing of the final test, participants performed better on the immediate test than on the delayed test, which was expected and consistent with the typically observed pattern of memory decline over time (Ebbinghaus, 1964; Murre & Dros, 2015). Importantly, the pretesting effect remained significant even with extended retention intervals, indicating that its benefits are not limited to the short-term but can also enhance long-term retention. This finding aligns with previous research using complex materials, such as trivia questions (Kornell, 2014) and prose texts (Kliegl et al., 2024), and extends the evidence to simpler, more controlled learning materials, such as weakly semantically related word pairs. Nevertheless, there was a slight decline in the magnitude of the pretesting effect with delayed final tests. This contrasts with the typical pattern observed in retrieval practice studies, where effects often become more pronounced with longer retention intervals (e.g., Roediger & Karpicke, 2006b). Although this divergence highlights potential differences in the underlying mechanisms of pretesting and retrieval practice, the similarities between the two, such as the benefits of retrieval-related processes and feedback, justify using the literature on the testing effect to inform predictions about pretesting. These similarities and differences emphasize the need for theoretical accounts that can explain the shared and unique aspects of these learning strategies, which warrant further investigation.

In addition to memory performance, we examined several other important aspects of the data that merit further discussion: error type analysis, reaction times during the final test phase, and metacognitive judgments. First, the error type analysis revealed distinctive patterns highlighting the potential differential influence of pretesting and reading on error processing. Four types of errors were analyzed: commission, omission, confusion, and intrusion errors. Notably, participants’ incorrect test responses were commission errors (incorrect responses that differ from initial errors). For omission errors (inability to produce any response), the read condition showed a higher incidence than pretesting, regardless of feedback timing, underscoring the advantage of pretesting in enhancing recall. These findings could indicate a more cautious and conservative response pattern when the material is learned under errorless reading conditions. Confusion errors (responses where participants select the target of a different cue) were more common in immediate than delayed testing, indicating that delaying the test may reduce interference effects. More importantly, intrusion errors (repeating initial learning-phase errors) did not differ according to feedback or test timing, suggesting minimal proactive interference from the errors made during pretesting. Our findings align with prior research (Knight et al., 2012; Wong & Lim, 2022; Maraver et al., 2022; Mera, Modirrousta-Galian et al., 2025) examining the effect of errorful learning conditions on intrusion errors. These studies consistently reported that commission errors were the most frequent error type. Together, these findings underscore the role of pretesting in mitigating error types that persist in passive learning conditions, potentially through error correction mechanisms and enhanced memory discrimination over time.

Second, reaction time analysis revealed mixed results, with significant differences observed only in Experiment 1, which did not extend to Experiment 2. In Experiment 1, learners responded faster in the read-only group when feedback was delayed, likely due to familiarity with the material learned without guessing. In contrast, participants in the pretest group had slower reaction times when feedback was delayed. Previous research has typically found that participants are slower to respond to items for which they had previously generated an error (Huelser & Metcalfe, 2012). This aligns with a phenomenon known as *Post-Error Slowing*, which indicates that committing errors slows down subsequent retrieval (Rabbitt & Rodgers, 1977). Post-error slowing is typically viewed as enhancing attention following errors (see Dutilh et al., 2012, for a review). These findings reveal that pretesting may foster more reflective and accurate recall, but this can be influenced by the timing of feedback. While our study focused on the latency to the first character typed as an indicator of initial retrieval, future research could examine total word typing time to provide a more comprehensive understanding of the entire retrieval process.

Finally, metacognitive judgments in our study revealed that although participants rated both conditions equally helpful, they underestimated their performance in the pretest but not in the read condition. Thus, participants were often unaware of the benefits of error generation during learning, leading them to underestimate their recall accuracy compared to their actual performance in the pretest condition. This phenomenon aligns with the concept of “metacognitive illusions” (e.g., Yan et al., 2016), where learners mistakenly view passive learning methods (such as reading) as more effective despite evidence to the contrary. This unawareness, or metamemory illusion, may stem from the straightforward nature of errorless learning techniques (such as reading), which can create a false sense of comprehension and retention. Consistent with prior findings, learning conditions facilitating fluent processing are often perceived as more effective than those that induce “desirable difficulties” (Bjork & Bjork, 2011). This pattern has been observed not only in studies on the pretesting effect (Huelser & Metcalfe, 2012; Yang et al., 2017) but also in research on retrieval practice (Kornell & Son, 2009) and lecture styles that vary in fluency (Carpenter et al., 2013). Nonetheless, these challenging, less intuitive methods often lead to superior long-term retention (Bjork & Bjork, 2011).

Our results align with the common metacognitive illusion in which fluent study conditions seem more effective, even though they are not objectively superior for learning. To mitigate this unawareness, providing self-regulation support, as suggested by recent research (Pan & Rivers, 2023), may help learners recognize the long-term advantages of errorful learning and pretesting, thus encouraging the adoption of these effective — albeit initially counterintuitive — learning strategies.

### Theoretical Implications

These findings have several important theoretical implications that contribute to our understanding of errorful learning. First, the results suggest the importance of immediate error correction in pretesting. This aligns with theories emphasizing the role of prediction error in learning (e.g., Brod, 2021; Brod et al., 2018; Grimaldi & Karpicke, 2012; Rescorla & Wagner, 1972), which suggests that learning is driven by the magnitude of the discrepancy between expected and actual outcomes. This reinforces the idea that immediate feedback would help learners adjust their predictions more effectively. Second, the observed stronger effects with immediate feedback and testing suggest that temporal contiguity between error generation, feedback, and testing may be crucial for maximizing the pretesting effect. This finding suggests that closeness in time between these factors can enhance learning outcomes. Third, the tendency to observe a diminishing effect with delayed testing contrasts with the typical patterns reported in retrieval practice studies, where benefits are often more visible over time. This divergence may imply that the mechanisms underlying pretesting and retrieval practice might differ. To further elucidate these theoretical implications, future research should explore whether the pretesting effect persists over longer retention intervals in both administering the final test and providing corrective feedback. It would also be valuable to investigate whether these findings generalize across different types of learning materials and diverse populations.

It is important to note that, while our findings demonstrate potential benefits of pretesting under controlled laboratory conditions, several limitations must be acknowledged. The study was conducted with a specific population (university students) and materials (semantically weak word pairs), which may not capture the complexity of real-world educational settings. As such, the generalizability of these results to diverse learners, learning materials, and real-world educational contexts remains uncertain. Rather than making direct claims about classroom applicability, we view our findings as contributing to the theoretical foundation needed to guide future applied research in more ecologically valid contexts.

## Conclusion

The results from two experiments demonstrate that the pretesting effect persists even with a long corrective feedback interval and when the final test is delayed. While the magnitude of the effect was reduced with feedback delays of 24 and 48 hours, it still remained significant. This finding contrasts with related phenomena, such as retrieval practice, where the effect tends to be stronger with longer retention intervals. The question remains whether the pretesting effect would persist over longer retention intervals. Importantly, the flexibility in timing makes pretesting particularly relevant for educational applications, where immediate feedback or testing may not always be feasible. These insights could inform strategies that maximize learning benefits even when logistical delays occur, supporting the value of pretesting as a versatile and effective approach for fostering durable learning.

## Data Accessibility Statement

All preregistrations, materials, data, and analytic codes needed to replicate this study are available on the OSF.

## Author Note

This work has been presented as a poster at the 45^th^ annual CogSci Conference and as two talks: at the XXXIV International Conference of the Spanish Society for Comparative Psychology (SEPC) and the XIV Conference of the Spanish Society for Experimental Psychology (SEPEX).
